# Characterization of the mechanisms underlying the crosstalk between galectins and notch in gastric cancer

**DOI:** 10.1186/1753-6561-7-S2-P65

**Published:** 2013-04-04

**Authors:** Sofia N Santos, Celso Reis, Marcelo Dias-Baruffi, Roger Chammas, Emerson S Bernardes

**Affiliations:** 1 Center for Translational Research in Oncology, Cancer Institute of the State of São Paulo, Brazil; 2 Carcinogenesis Group, Institute of Molecular Pathology and Immunology of the University of Porto, Portugal; 3 Faculty of Pharmaceutical Sciences of Ribeirão Preto, University of São Paulo, Brazil

## Background

Gastric cancer is the fourth most common cancer and the second leading cause of cancer-related deaths worldwide. Galectins form a family of β-galactosides binding proteins that recognize a variety of glycan-containing proteins at the cell surface and are overexpressed in various tumors, including gastric cancer. Galectins overexpression as well as changes in their subcellular distribution has been associated with gastric cancer progression and poor prognosis. It is not well understood, however, how the interaction between galectins and glycosylated receptors modulates tumor development and growth. Since Notch receptors and ligands contain glycan structures known to bind galectins, we aim to demonstrate that galectins expression in the tumor microenvironment may interfere with Notch signaling activation during tumor development and progression.

## Materials and methods

Immunoprecipitation procedures with gastric cancer cell line AGS (ATCC CRL-1739) and MKN45 (ACC 409) were used to test for association between galectin-1/-3 and Notch-1 receptor. Furthermore, we transfected AGS cell line with siRNA against galectin-1/-3 or scramble using standard protocols (IDT DNA technologies), stimulate them with immobilized human recombinant delta-4 or Jagged-1 and assessed Notch-1 receptor activation.

## Results

Galectin-1 and -3 interact with Notch-1 receptor and differentially modulate Notch signaling pathway upon activation by Delta/Jagged ligands. Galectin-1 knockdown alters Notch-1 activation induced by Delta-4 whereas galectin-3 knockdown alters jagged-1-mediated Notch-1 activation. Furthermore, we found that exogenously added galectin-3 can enhance Notch-1 activation by Jagged-1.

## Conclusion

Our results suggest that galectin-1 and -3 interact with Notch-1 receptor and differentially modulate Notch signaling activation induced by Jagged-1 and Delta-4.

## Financial support

FAPESP and CAPES.

